# Results of a German nationwide survey on perioperative cardiac management in vascular surgery

**DOI:** 10.1007/s00423-024-03523-5

**Published:** 2024-11-12

**Authors:** Dmitriy I. Dovzhanskiy, Moritz S. Bischoff, Karola Passek, Dittmar Böckler

**Affiliations:** Department of Vascular and Endovascular Surgery, University Hospital Heidelberg, Im Neuenheimer Feld 110, 69120 Heidelberg, Germany

**Keywords:** Vascular surgery, Cardiac risk stratification, Perioperative myocardial ischemia, Cardiac management, Survey

## Abstract

**Abstract:**

Because of the lack of specific recommendations concerning cardiac risk stratification before vascular surgery, appropriate decisions remain individual. The aim of the present study was to evaluate the perioperative cardiac management in vascular surgery in Germany.

**Methods:**

This article is based on a survey from 2018 of heads of German vascular surgical departments or units regarding their experience with perioperative cardiac management. The questionnaire asked about the experience with preoperative cardiac evaluation and its extension, awareness of perioperative myocardial ischemia, the art of postoperative monitoring and the routine use of the best medical treatment.

**Results:**

In total, 62% of responders agreed that perioperative myocardial ischemia is a relevant postoperative problem in their clinic after open abdominal aortic surgery, while 47% stated the same after vascular surgery (VS) like carotid endarterectomy, peripheral arterial surgery or EVAR. Preoperative cardiological evaluations are performed routinely by 87% of responders before open abdominal aortic surgery and by 42% before VS. Preoperative cardiac evaluation included cardiac echography in 92% and stress diagnostics (stress echography, stress ECG) in 38%. Routine preoperative cardiac catheterisation is performed in 4% before OAS and only 0.5% before VS. In addition, 79% of participants initiate acetylsalicylic acid routinely and 68% use statins preoperatively. The serum troponin diagnostic test in asymptomatic patients was routinely applied by 19% of responders after OAS and by 6% after VS.

**Conclusion:**

Perioperative myocardial ischemia is considered a relevant problem, primarily after aortic surgery. The preoperative cardiac stress diagnostics among vascular surgeons does not seem to be sufficiently widespread. The preoperative initiation of acetylsalicylic acid and statins is not routine in 30% of hospitals.

## Introduction

Perioperative myocardial ischemia is a serious complication after vascular surgery [1]. This implies that risk stratification should be applied for any patient [2]. The task of risk evaluation aims to preoperatively identify patients with a previously unrecognised or insufficiently treated disease that is relevant to surgery or anaesthesia and to optimise therapy. Preoperative history and physical examination are accepted standards in the risk evaluation of patients before elective surgical procedures. Whether and under what circumstances technical preliminary examinations can help to reduce the perioperative risk has so far been insufficiently investigated [3].

High co-morbid vascular surgery patients are at an increased risk of cardiovascular complications [4]. In particular, patients who undergo conventional aortic surgery are especially affected because of essential cross-clamping [5].

The question of whether planned vascular surgery is actually feasible in elderly comorbid patients is part of everyday clinical practice. It is not always possible to consult a cardiologist in the short-term for time or organisational reasons. Therefore, a vascular surgeon himself often decides whether to carry out additional preoperative examination or not. Some questions concerning risk stratification are well known, for example the need to consider initiating statins perioperatively if indicated [6]. Other questions are less defined. For example, what is the appropriate extension of preoperative cardiac evaluation? Which patients should be consulted by a cardiologist preoperatively? Is cardiac echography alone enough? Are stress diagnostics (like stress echography or stress ECG) or preoperative cardiac catheterisation in the case of multisite artery disease indicated? Another question is how to deal with vascular patients’ post-surgery. Which individuals need routine postoperative monitoring and for how long? The last question mostly depends on the local standard operating procedures.

Thus, there are still no specific recommendations for cardiac risk stratification concerning vascular surgery patients and the decision remains individual. Therefore, the aim of the present study was to evaluate the perioperative cardiac management in vascular surgery in Germany.

## Materials and methods

In 2018, a questionnaire was sent by post to 324 identified vascular surgery departments or units in Germany. These were identified by means of internal databases and the website www.kliniken.de.

The questionnaire asked about experience with preoperative cardiac evaluation and its extension, awareness of perioperative myocardial ischemia, the art of postoperative monitoring and the routine use of the best medical treatment. Additionally, the questionnaire asked about the characteristics of the participating clinics: the organisational structure of the vascular surgery incl. bed capacity and the presence of a cardiac catheterisation laboratory in the hospital. Results are presented descriptively.

Categorical variables were analysed with Fisher’s exact test. Microsoft Office Excel 2013 (Microsoft, Redmond, USA) and GraphPad Prism (version 5 for Windows; GraphPad Software, Inc., San Diego, CA, USA was used for analysis.

## Results

**Fig. 1:**
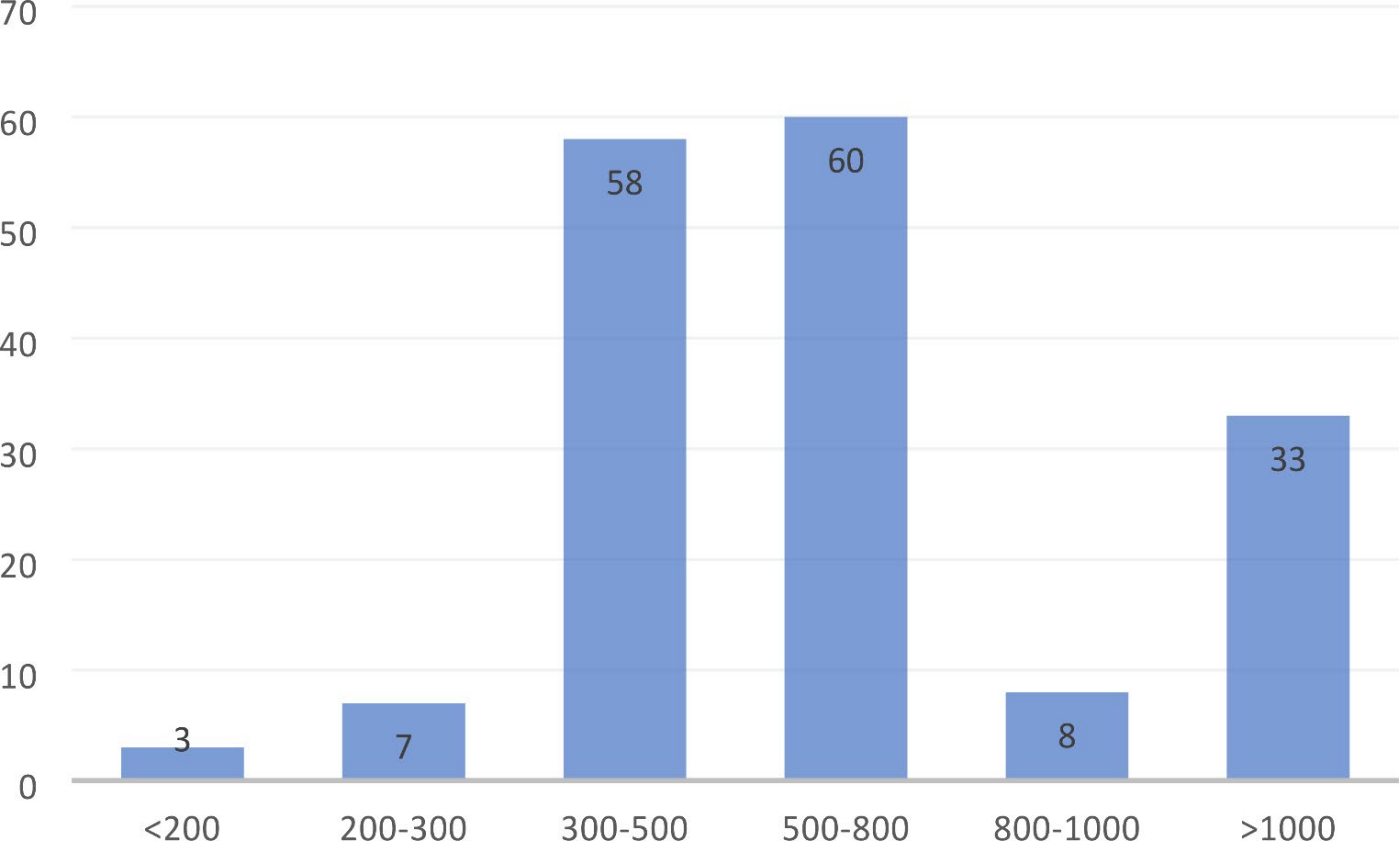
Bed capacity of participating hospitals

**Fig. 2:**
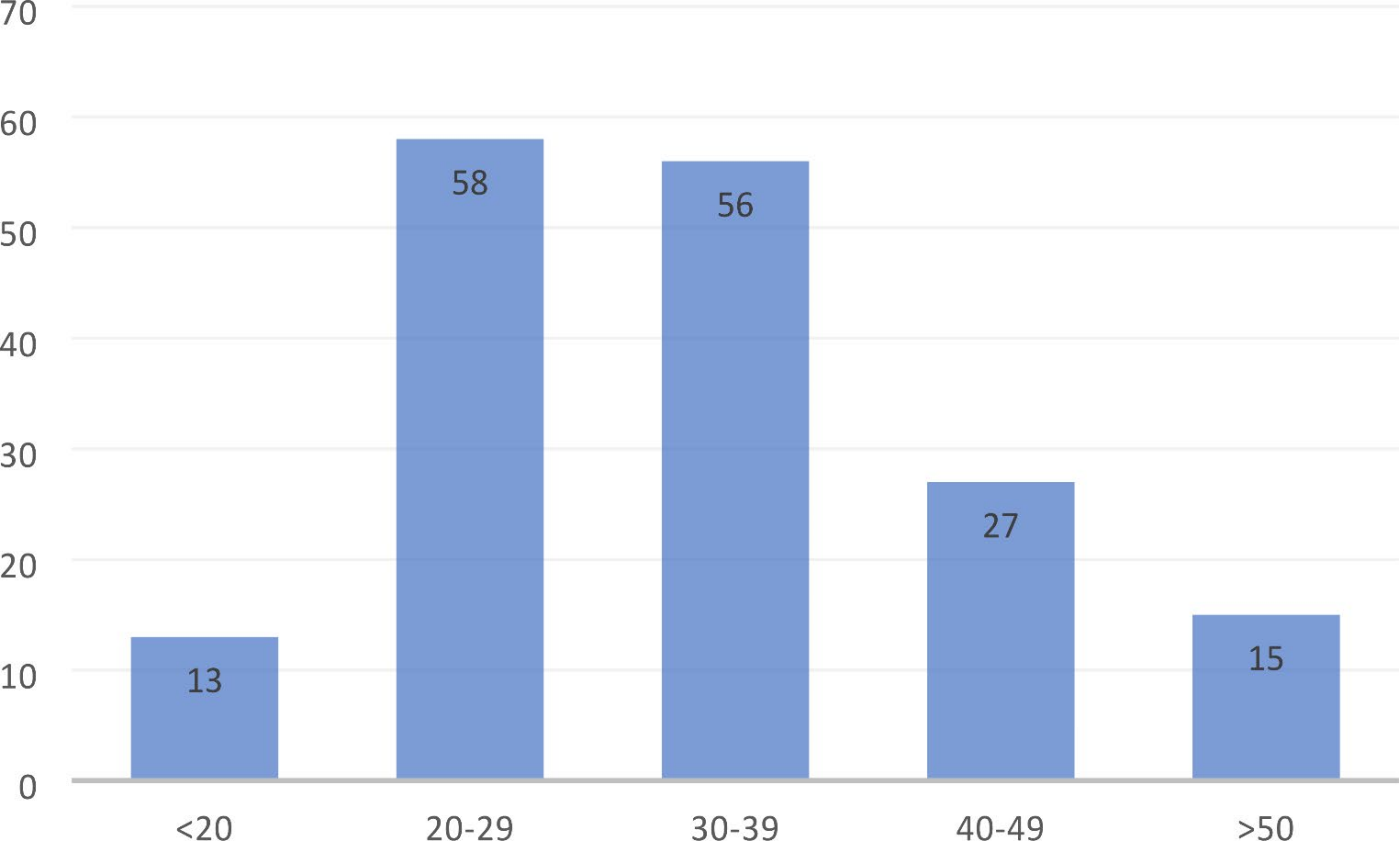
Bed capacity of vascular surgery

**Fig. 3:**
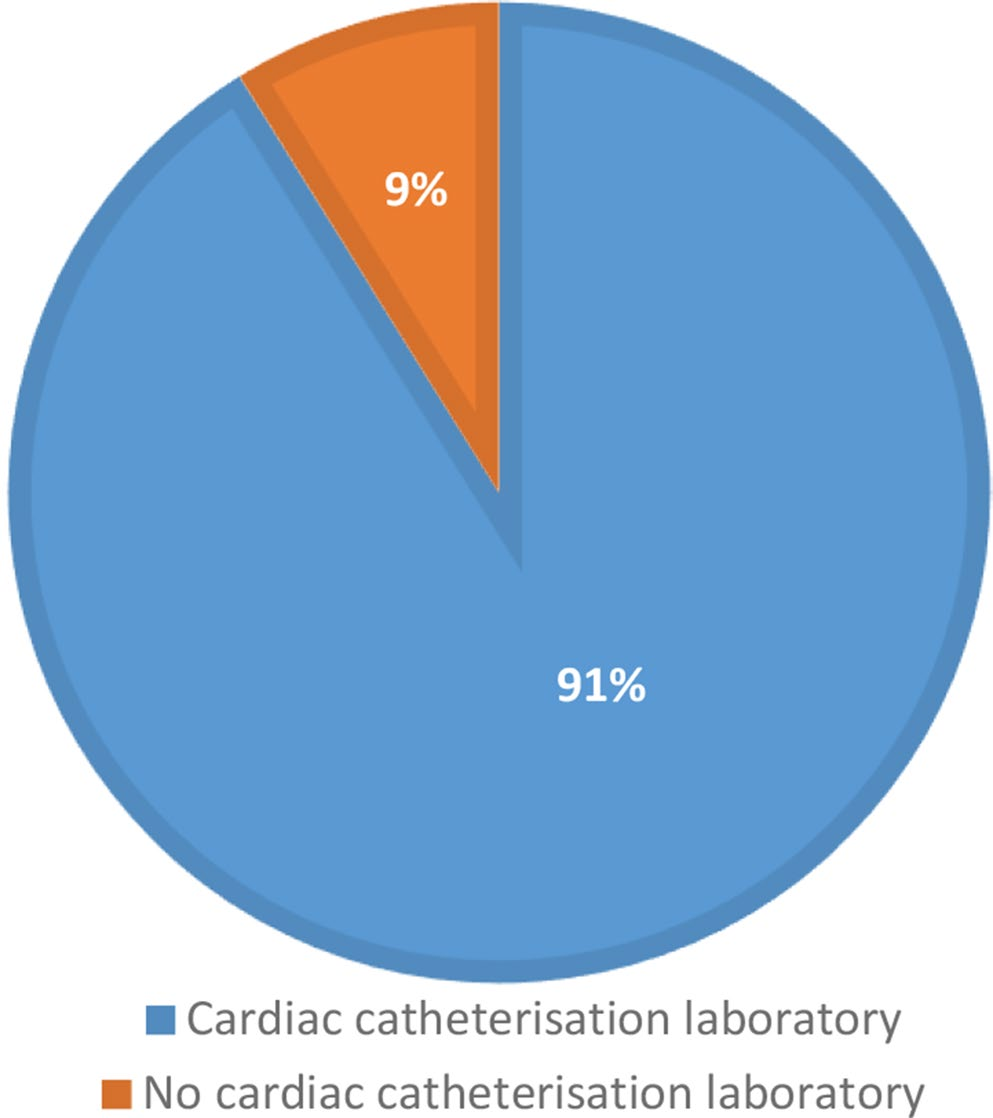
Availability of a cardiac catheterization laboratory

**Fig. 4:**
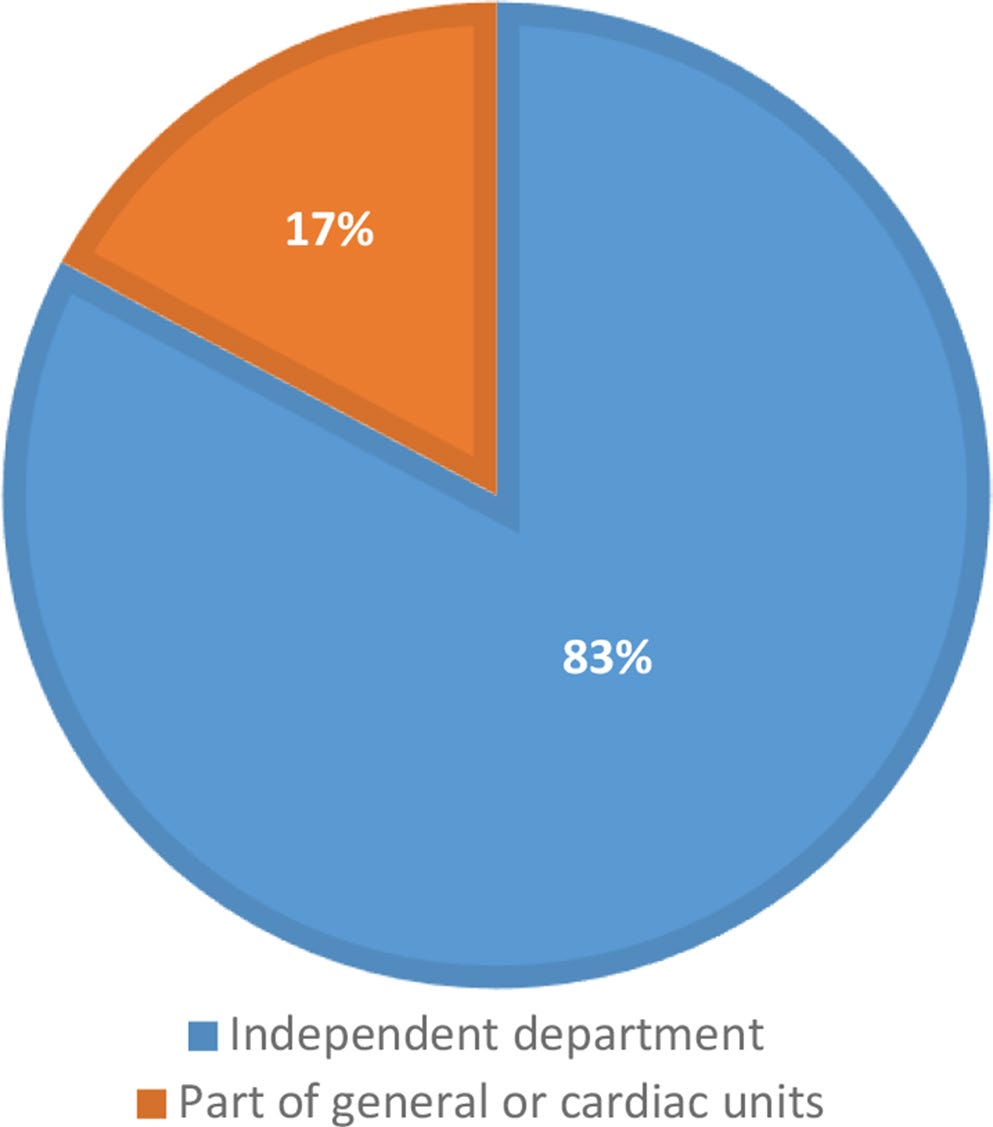
Structure of vascular surgery

The response rate was 52% (169/324). The median bed capacity of participating hospitals was 562, with a range of 156–2600 (Fig. 1). The median bed capacity of vascular surgery was 30 with a range between 10 and 85 (Fig. 2). Overall, 154 general or cardiac hospitals (91%) had a cardiac catheterisation laboratory in their structure (Figs. 3) and 140 (83%) respondents reported independent departments of vascular surgery, while the remaining respondents were parts of surgery units (Fig. 4).

The majority (62%) of responders agreed that perioperative myocardial ischemia would be a relevant postoperative problem in their clinic after open abdominal aortic surgery, 47% agreed the same after vascular surgery (VS) such as carotid endarterectomy, peripheral arterial surgery or EVAR and 22% in the case of peripheral intervention (PI) because of peripheral artery disease (PAD). Overall, 79% of participants initiate acetylsalicylic acid routinely, while 68% use statins and 30% β-blockers before arterial surgery.

Preoperative cardiological evaluations are performed routinely by 87% of responders before open abdominal aortic surgery, by 42% before VS and by 12% before PI. The preoperative cardiac evaluation included cardiac echography in 92% and stress diagnostics (stress echography, stress ECG) in 38%. Routine preoperative cardiac catheterisation is performed in 4% before OAS and 0.5% before VS.

After OAS, 14% of participants perform routine postoperative monitoring in the setting of an intensive care unit for less than 24 h, 38% for 24 h, 37.5% for 24–48 h, 10% for 72 h and 0.5% for 96 h (Tables 1, 2, 3, 4 and 5).

**Table 1:** Do you consider perioperative myocardial ischemia to be a relevant postoperative problem in your clinic?

Open abdominal aortic surgery	103 (62%)
Further vascular surgery (carotid endarterectomy, peripheral arterial surgery	78 (47%)
Peripheral Intervention because of PAD	37 (22%)

**Table 2:** Do you perform preoperative cardiological evaluations routinely in your clinic?

Before open abdominal aortic surgery	144 (87%)
Before further vascular surgery (carotid endarterectomy, peripheral arterial surgery	69 (42%)
Before peripheral Interventions because of PAD	20 (12%)

**Table 3:** What does a preoperative cardiac evaluation include?

cardiac echography	156 (92%)
Stress diagnostics (stress echography, stress ECG)	45 (38%)
Miscellaneous	19 (4%)

**Table 4:** Does your clinic routinely perform preoperative cardiac catheterization?

Before open adbominal aortic surgery	7 (4%)
Before further vascular surgery (carotid endarterectomy, peripheral arterial surgery	1 (0.5%)

**Table 5:** Do you routinely use the following medications in your clinic before vascular surgery?

Acetylsalicylic Acid	133 (79%)
Statins	115 (68%)
β-Blocker	51 (30%)

**Table 6:** How long do you perform routine postoperative monitoring in the intensive care unit after open abdominal aortic surgery?

< 24 h	20 (14%)
24 h	55 (38%)
24–48 h	54 (37.5%)
72 h	14 (10%)
96 h	1 (0,5%)

The duration of postoperative monitoring in the intensive care unit after EVAR, peripheral artery surgery and PI is shown in Tables 6,7 and 8. After EVAR, 20% do not perform any intensive care monitoring, 10% perform less than 6 h, 23% perform monitoring between 12 and 24 h and 45% for 24 h. About half (48%) of responders do not monitor patients after peripheral arterial surgery, 14% monitor them for less than 6 h, another 14% for between 12 and 24 h and 22% for 24 h. The most percipients (89%) don’t monitor the patients after PI.

**Table 7:** How long do you perform routine postoperative monitoring in the intensive care unit after further vascular surgery in your clinic?

	Endovascular Aortic Repair (EVAR)	Peripheral arterial surgery	Peripheral interventions because of PAD
0 h	33 (20%)	77 (48%)	146 (89%)
< 6 h	17 (10%)	23 (14%)	11 (7%)
12–24 h	38 (23%)	22 (14%)	7 (4%)
24 h	75 (45%)	35 (22%)	
24–48 h		2 (2%)	
72 h	2 (2%)		

**Table 8:** Does your clinic routinely measure postoperative troponin (in asymptomatic patients)?

After open abdominal aortic surgery	32 (19%)
After further vascular surgery (carotid endarterectomy, peripheral arterial surgery	10 (6%)

The serum troponin diagnostic test in asymptomatic patients was routinely applied postoperatively by 19% of responders after OAS and by 6% after VS.

The availability of cardiac catheterization laboratory didn’t influence the protocols of participants in a case of open abdominal aortic surgery. There were no significant differences between the clinics with or without catheterisation laboratory in preoperative cardiological evaluations (*p* = 0.12), in routine preoperative cardiac catheterization (*p* = 0.13) and in routine measurement of postoperative troponin (*p* = 0.74).

## Discussion

Cardiac risk stratification remains a task in the preoperative routine. The recent German [3] and European [6] guidelines describe cardiac risk stratification before non-cardiac or non-cardiothoracic surgery, but there is a lack of specific recommendations concerning vascular surgery. Therefore, the risk stratification for these patients is usually performed individually.

The preoperative evaluation should be carried out at a sufficient interval from the surgical procedure, as this can reduce the duration of the inpatient stay, the number of cancelled operations and costs [3, 7, 8]. It would be desirable to schedule the necessary preliminary examinations at the time of the indication for surgery, although an interval of 6 weeks between evaluation and surgery should not be exceeded [9]. The relative time pressure in often urgent cases of vascular diseases (like critical limb ischemia, symptomatic carotid stenosis or large aortic aneurysms) can complicate the decision. Consultation by cardiologists could be helpful in clarifying risk stratification. Nowadays, the organisation of short-term cardiological consultation may be challenging because of the intensification of clinical routine. Nevertheless, preoperative cardiological evaluations are performed routinely by 87% of responders before OAR and 42% before VS in our study.

Perioperative myocardial ischemia is a serious and relevant postoperative complication. A significant proportion of responders in our study (62% after OAR, 47% after VS and 22% after PI) agree with this statement for their clinics. At the same time, the diagnosis of perioperative myocardial ischemia is not uniform. The difference between myocardial infarction and myocardial injury was described in the Fourth Universal Definition of Myocardial Infarction. In addition, the term perioperative myocardial injury was defined as an extra category of myocardial ischemia [10], whereas the most important factor here is an increase in cardiac troponin without clinical or electrocardiographic evidence of acute myocardial ischemia. The routine measurement of cardiac troponin postoperatively is not established in everyday clinical practice after non-cardiac surgery. Therefore, subclinical myocardial injury with potentially serious consequences remains undetected in many cases. Although routine troponin measurement leads to a certain increase in costs, this procedure has been recommended for high-risk patients [11]. Otherwise, an asymptomatic increase in troponin is associated with a worsening of medium- and long-term mortality [12, 13]. The results of our own working group have also shown a significant worsening of long-term survival in patients with asymptomatic myocardial ischemia after aortic surgery [14]. In the present study, the routine measurement of troponin has been performed postoperatively in only 19% of clinics after OAR and in 6% after VS.

In cases where cardiac evaluation has been indicated, the next questions appear to be about its extent. Is it possible to stratify cardiac risk only by means of patient history and physical examination? If it will not be enough, what kind of additional examination should it be? In a recent study, preoperative cardiac evaluation included cardiac echography in 92% and stress diagnostics (stress echography, stress ECG) in 38%; routine preoperative cardiac catheterisation would be performed in 4% before OAS and only 0.5% before VS.

The assessment of perioperative cardiac risk and the decision for or against extended preoperative diagnostics are based essentially on 4 factors: (a) the presence of acutely symptomatic heart disease, (b) the cardiac risk of the surgical procedure, (c) the presence of cardiac risk factors and (d) the patient’s physical resilience [3, 6, 9, 15]. Patients with known coronary artery disease or a high risk of ischemia should be evaluated by a multidisciplinary treatment team consisting of a surgeon, anaesthesiologist and cardiologist before a planned high-risk procedure [15]. The concepts for the preoperative evaluation of adult patients before operations represent interdisciplinary recommendations with the goal of ensuring a high level of patient orientation while avoiding unnecessary examinations, shortening preoperative examination processes and ultimately reducing costs [9]. However, this also means that individual concepts must be created for individual patients. Adequate physical resilience is an excellent predictor of a good perioperative outcome, which is why additional preoperative examinations are rarely indicated in such patients [9]. The preoperative 12-lead ECG is intended to detect cardiac diseases that influence the anaesthesiologic procedure [9]. Preoperative echocardiography is only recommended before noncardiac surgery in patients with new-onset dyspnoea of unknown origin and in patients with known heart failure and the worsening of symptoms within the last 12 months [9]. From another side, it does not make a decisive contribution to perioperative risk reduction in cases of known, stable heart failure [16]. The benefit of routine preoperative resting echocardiography in normal patients before high-risk procedures is unclear [15]. The use of stress imaging is appropriate for risk assessment in patients with clinical risk factors and poor functional capacity [17, 18]. Stress imaging is recommended before high-risk elective surgery in patients with poor functional capacity and a high likelihood of or high clinical risk. Moreover, it should be considered before high-risk surgery in asymptomatic patients with poor functional capacity, and previous percutaneous coronary intervention or coronary artery bypass graft [6]. Concerning invasive coronary diagnostics (ICA), there is a lack of information from randomised controlled trials relating to the usefulness of it in patients scheduled for non-coronary surgery. Adopting an ICA assessment may also cause an unnecessary and unpredictable delay in an already planned surgical intervention and adding an independent procedural risk to the overall risk. The indications for preoperative coronary angiography and revascularisation are similar to angiography indications in the non-surgical setting. The preoperative treatment of patients with myocardial ischaemia, either medically or with intervention, is recommended [6].

The control of cardiovascular risk factors, including arterial hypertension, dyslipidaemia, and diabetes, is important before non-cardiac surgery [6]. The ACC/AHA guidelines recommend that low-dose aspirin use should be considered for the primary prevention of atherosclerotic cardiovascular disease among select adults at higher cardiac risk but not at increased risk of bleeding [19]. In patients with a previous PCI, it is recommended to continue aspirin peri-operatively if the bleeding risk allows [6]. In patients with an indication for statins, it should be considered to initiate statins perioperatively. The question about the pre-surgery initiation of beta-blockers has been a matter of intense controversy. The routine initiation of beta-blockers perioperatively is not recommended. Nevertheless, the pre-operative initiation of beta-blockers in advance of non-cardiac surgery may be considered in patients who have known coronary artery disease or myocardial ischaemia [6]. In a recent study, 79% of participants routinely used acetylsalicylic acid, 68% used statins and 30% used β-blockers before arterial surgery.

We did not find any specific recommendations concerning the duration of postoperative monitoring in the intensive care unit after vascular surgery. Only in the guidelines of the Society of Vascular Surgery was postoperative management in an ICU recommended for patients with significant cardiac, pulmonary, or renal disease as well as for those requiring postoperative mechanical ventilation or who developed a significant arrhythmia or haemodynamic instability during operative treatment [20]. In a recent study, the duration of postoperative monitoring was very different between responders.

According to results of this study, the awareness of cardiac risk stratification should be underlined in the daily praxis. The assessment of perioperative cardiac risk and the decision for or against extended preoperative diagnostics is a task for a multidisciplinary treatment team. The postoperative routine troponin measurement should be considered by high-risk patients. The duration of postoperative monitoring in the intensive care unit after vascular surgery is not clarified and be evaluated in further studies.

This study has several limitations. It is a questionnaire-based work, which depends on the care of the respondents. The participants belonged to different levels of the hospital service, the questionnaires have also not been evaluated and the response rate was 52%. Finally, the study population is heterogeneous with respect to vascular pathologies and procedures performed.

## Conclusion

Perioperative myocardial ischemia is considered a relevant problem, primarily after aortic surgery. The preoperative cardiac stress diagnostics among vascular surgeons does not seem to be sufficiently widespread. The preoperative initiation of acetylsalicylic acid and statins is not routine in 30% of hospitals. There is a high degree of heterogeneity in the duration of postoperative intensive care monitoring.

## Data Availability

No datasets were generated or analysed during the current study.
